# Primary pulmonary melanoma: a report of two cases

**DOI:** 10.1186/s12957-015-0695-2

**Published:** 2015-09-17

**Authors:** Mototsugu Watanabe, Hiromasa Yamamoto, Shinsuke Hashida, Junichi Soh, Seiichiro Sugimoto, Shinichi Toyooka, Shinichiro Miyoshi

**Affiliations:** Department of Thoracic, Breast and Endocrinological Surgery, Okayama University Graduate School of Medicine, Dentistry and Pharmaceutical Sciences, Okayama, 700-8558 Japan; Department of Clinical Genomic Medicine, Okayama University Graduate School of Medicine, Dentistry and Pharmaceutical Sciences, 2-5-1 Shikata-cho, Kita-ku, Okayama 700-8558 Japan; Biobank of Okayama University Hospital, Okayama, 700-8558 Japan

**Keywords:** Primary pulmonary malignant melanoma, Target sequencing, Surgical resection

## Abstract

Malignant melanoma is a refractory malignancy with a dismal prognosis. It generally arises from the skin in most cases, and cases of primary pulmonary malignant melanoma are rare and often behave aggressively. We have treated two cases of localized primary pulmonary malignant melanoma using surgical resection. Pulmonary malignant melanomas often metastasize to the brain and liver; one of our cases exhibited metastasis to the cecum at about 8 months after surgery. Because cutaneous melanomas often carry activating mutations in the *BRAF* gene (V600E), we performed a *BRAF* mutational analysis using direct sequencing for both of these tumors arising from the lung. However, no *BRAF* mutations were detected. We detected a p53 mutation, which was thought to be a potential somatic mutation, in one of the two cases using a sequencing panel targeting 20 lung cancer-related genes. Although we also checked the expression of programmed death ligand 1 (PD-L1) on the surface of the tumor cells by immunohistochemical testing, neither of our two cases expressed PD-L1. Further molecular analyses may uncover the characteristics of primary pulmonary malignant melanomas.

## Background

Malignant melanoma is an often fatal cutaneous neoplasm. As a primary tumor, malignant melanoma of the lung is rare, accounting for only 0.01 % of all primary lung tumors [1]. To diagnose malignant melanoma as a primary tumor arising from the lung, the absence of primary melanoma lesions at cutaneous or nonpulmonary extracutaneous sites must be demonstrated [2].

Approximately 40 to 60 % of cutaneous melanomas carry mutations in the *BRAF* gene (V600E), which leads to the constitutive activation of downstream signaling through the MAPK pathway [3, 4]. At present, whether pulmonary malignant melanomas carry *BRAF* mutations has not been reported. Additionally, immunotherapy to inhibit programmed death 1 (PD-1)/programmed death ligand 1 (PD-L1) pathway that has a role for immune escape is developed for malignant melanoma [5, 6]. It is reported that the efficacy of anti-PD-1 antibody was related to the expression of PD-L1 in tumor cells [6]. Here, we describe two cases of possible primary pulmonary malignant melanoma with wild-type *BRAF* and no expression of PD-L1.

## Case presentation

### Case 1

**Fig. 1:**
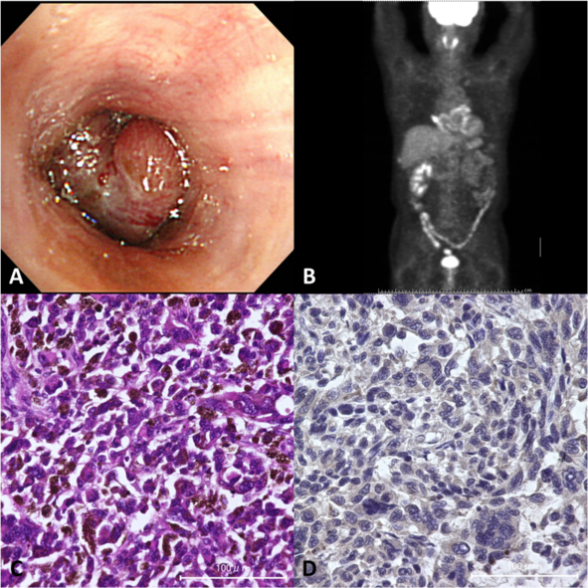
Diagnostic images and pathological findings for case 1. **a** A bronchoscopy revealed a tumor arising from the right middle lobe bronchus. **b** PET-CT scan revealed no other lesions that could have represented a primary site. **c** Hematoxylin-eosin staining of the resected tumor, ×40 (*scale bar*, 100 μm). **d** Immunohistochemistry showed negative for PD-L1 staining, ×40 (*scale bar*, 100 μm)

A 66-year-old man with hemoptysis was referred to our hospital because of a pulmonary malignant tumor. A bronchoscopy revealed a tumor arising from the right middle lobe bronchus (Fig. 1a), and a histological diagnosis based on a bronchial biopsy revealed a malignant melanoma. A positron emission tomography-computed tomography (PET-CT) scan revealed no other lesions that could have represented a primary site (Fig. 1b). A right middle lobectomy with mediastinal and hilar lymphadenectomy was performed. Some tumor cells had melanin in the cytoplasm (Fig. 1c). Immunohistochemical analysis showed positive cytoplasmic staining of the tumor cells for HMB-45, Tyrosinase, and MART1, although the expression of TTF-1 in tumor cells was negative. The postoperative course was uneventful. Combination chemotherapy of dacarbazine, nimustine, and vincristine was performed. At the time of the 22 months after surgery, he was alive without disease recurrence.

### Case 2

**Fig. 2:**
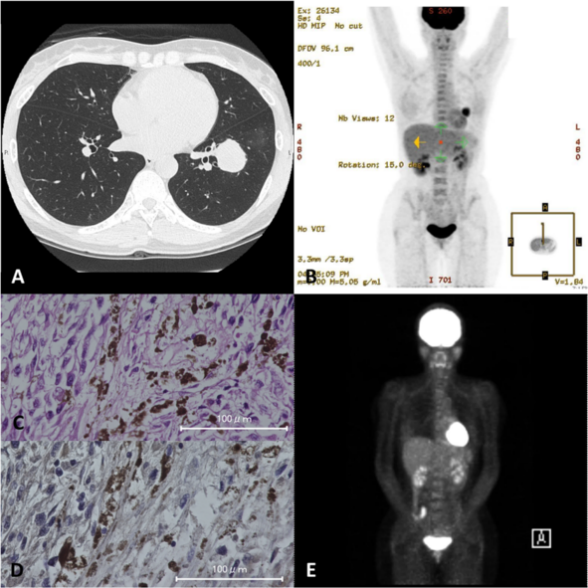
Diagnostic images and pathological findings for case 2. **a** A CT scan shows a large tumor in the left lower lobe of the lung. **b** Preoperative PET-CT scan revealed no other lesions that could have represented a primary site. **c** Hematoxylin-eosin staining of the resected tumor, ×40 (*scale bar*, 100 μm). **d** Immunohistochemistry showed negative for PD-L1 staining, ×40 (*scale bar*, 100 μm). **e** Eight months after the pulmonary resection, PET-CT scan showed the uptake of fluorodeoxyglucose in the cecum

A 46-year-old woman was found to have an abnormal shadow on a chest X-ray during an annual check-up. A CT and PET-CT scan showed a tumor in the left lower lobe of the lung (Fig. 2a, b). Pathological examination of a specimen obtained during a transbronchial lung biopsy revealed that the tumor was a malignant melanoma (Fig. 2c). Because further examinations did not reveal any other tumors that could have been the primary lesion, we diagnosed the lung tumor as a primary pulmonary malignant melanoma. A left lower lobectomy with mediastinal and hilar lymph node dissection was performed. Immunohistochemical staining was negative for S-100 protein, chromogranin, synaptophysin, keratin, and TTF-1 but positive for HMB-45 and vimentin. The postoperative course was uneventful. She refused to receive adjuvant chemotherapy. Eight months after the pulmonary resection, a PET-CT scan showed the uptake of fluorodeoxyglucose in the cecum (Fig. 2e). An ileocecal resection was performed, and the pathological examination indicated that the tumor was considered to be the recurrence of a melanoma.

### Molecular profiling

We determined the *BRAF* mutational status (exons 11 and 15) using direct sequencing in the two tumors. The detailed methods have been described previously [7]. However, we did not detect any *BRAF* mutations. Next, we performed a target sequencing analysis using the Human Lung Cancer Panel (Qiagen, Hilden, Germany), which targets 20 lung cancer-related genes including most of the exons in *BRAF*, using the same samples. Although various variants were detected using this analysis, only the p53 mutation (P72R) in case 1 was considered to be a potential somatic mutation after restricting the variants using GeneRead Software (Qiagen, Venlo, Netherlands) as shown in Table 1. P72R mutation has been already reported in bladder and gastric cancers, but there are no reports in lung cancer or malignant melanoma ([8], accessed on August 14, 2015).

**Table 1:** Mutational status of 20 genes in cases 1 and 2

Gene name	Case 1	Case 2
*MTOR*	WT	WT
*NRAS*	WT	WT
*PTGS2*	WT	WT
*ALK*	WT	WT
*CTNNB1*	WT	WT
*PIK3CA*	WT	WT
*PDGFRA*	WT	WT
*KIT*	WT	WT
*EGFR*	WT	WT
*MET*	WT	WT
*BRAF*	WT	WT
*CDKN2A*	WT	WT
*PTEN*	WT	WT
*HRAS*	WT	WT
*KRAS*	WT	WT
*RB1*	WT	WT
*AKT1*	WT	WT
*TP53*	P72R	WT
*ERBB2*	WT	WT
*STK11*	WT	WT

*WT* wild type

Furthermore, we also checked the expression of PD-L1 on the surface of the tumor cells by immunohistochemical testing in formalin-fixed, paraffin-embedded tumor specimens with the use of a rabbit monoclonal antihuman PD-L1 antibody. In the previous reports, PD-L1 positivity was defined as at least 5 % of tumor cells showing cell-surface PD-L1 staining of any intensity in a section containing at least 100 tumor cells that could be evaluated [9, 10]. According to the definition of PD-L1 positivity by them, neither of our two cases was considered to express PD-L1 (Figs. 1d and 2d).

## Discussion

The total number of operations in general thoracic surgery is increasing year by year in Japan [11]. However, there are a few reports about primary pulmonary melanoma [2]. There are four clinical criteria that should be satisfied for the diagnosis of primary melanoma of the lung: (1) no previously removed skin tumor, unless the pathology examination did not show malignancy and the slides are still available for reevaluation; (2) no excised ocular tumor; (3) a solitary tumor in the surgical specimen from the lung; and (4) no demonstrable melanoma in other organs at the time of surgery [1, 12]. Both of our cases fulfill the abovementioned diagnostic criteria.

Regarding treatment, surgery is necessary for a cure for localized pulmonary malignant melanoma because the effect of chemotherapy on malignant melanoma is limited [13]. Even among patients who undergo surgery, the prognosis of patients with malignant melanoma of the lung is generally poor because the lesion often metastasizes soon after surgery. Regarding metastatic spread, primary pulmonary melanomas often metastasize to the brain and the liver [2, 14–16], similar to other types of primary lung cancer. Intriguingly, one of our cases exhibited metastasis to the cecum. To the best of our knowledge, this is the first report describing a malignant melanoma of the lung metastasizing to the cecum.

*BRAF*-activating mutation is a characteristic of malignant melanoma, and molecular-targeted therapy for BRAF has been developed [17]. However, our two cases did not exhibit activating mutations in *BRAF*. Considering the fact that the presence of *BRAF*-activating mutations has not been reported in pulmonary malignant melanoma, their features may differ from those of primary skin melanoma. A target sequencing analysis did not detect any possible somatic driver mutations commonly observed in lung cancer [18], such as *EGFR*, *HER2*, *KRAS*, *ALK*, *MET*, or *PIK3CA*, suggesting that primary pulmonary malignant melanomas may have different oncogenic pathways from primary lung cancer from the viewpoint of driver oncogenes.

In these days, there are many reports about the efficacy of anti-PD-1 antibody for cutaneous melanoma patients and a potential association between the occurrence of a response and expression of PD-L1 in patients receiving nivolumab [10]. On the other hand, Robert et al. reported that regardless of PD-L1 status, nivolumab-treated patients had improved overall survival, as compared with dacarbazine-treated patients [19]. Although our findings did not show the positivity of PD-L1 in the two cases, there is still the possibility of the efficacy of immunotherapy for primary pulmonary melanoma, as the role of PD-1/PD-L1 pathway in primary pulmonary melanoma is yet to be investigated.

## Conclusions

In conclusion, we encountered two cases of malignant melanoma of the lung that did not carry activating mutations in the *BRAF* gene. Further molecular analyses may uncover the characteristics of primary malignant melanoma.

## Consent

Written informed consent was obtained from the patients for publication of this case report and any accompanying images. A copy of the written consent is available for review by the editor in chief of this journal.

## Abbreviations

PD-1: Programmed death 1
PD-L1: Programmed death ligand 1
PET-CT: Positron emission tomography-computed tomography
